# Editorial: Artificial Intelligence for Precision Medicine

**DOI:** 10.3389/frai.2021.834645

**Published:** 2022-01-21

**Authors:** Jun Deng, Thomas Hartung, Enrico Capobianco, Jake Y. Chen, Frank Emmert-Streib

**Affiliations:** ^1^Department of Therapeutic Radiology, Yale University, New Haven, CT, United States; ^2^Bloomberg School of Public Health, Johns Hopkins University, Baltimore, MA, United States; ^3^Institute of Data Science and Computing, University of Miami, Coral Gables, FL, United States; ^4^National Research Council of Italy (CNR), Institute of Organic Synthesis and Photoreactivity, Bologna, Italy; ^5^University of Alabama at Birmingham, Birmingham, AL, United States; ^6^Predictive Society and Data Analytics Lab, Faculty of Information Technology and Communication Sciences, Tampere University, Tampere, Finland

**Keywords:** precision medicine, individualized healthcare, artificial intelligence, big data, machine learning

## Scope and Aim of This Research Topic

Fueled by advances in computing power, algorithms, and big data, the last decade has witnessed widespread applications of artificial intelligence (AI) in every major field, including medicine and healthcare. Generally speaking, AI is expected to help realize the promise of precision medicine in three major areas: (1) disease prevention, (2) personalized diagnosis, and (3) personalized treatment. In this Research Topic, “Artificial Intelligence for Precision Medicine,” we aim to set up an open stage in the community where breakthrough application examples of AI for precision medicine are presented. We envisage that AI technologies, if applied openly, fairly, robustly, and in close collaboration with human intelligence, will open new doors for effective and personalized healthcare worldwide.

## Topics Covered in This Research Topic

- AI-aided diagnosis and early detection of diseases: Hart et al.
- AI-enhanced treatment and delivery: Chen et al.; Jensen et al.; Mistro et al.; Wang et al.
- Clinical decision support with AI techniques: Barua et al.
- Enhancing patient care via AI applications: Luo
- Radiomics and quantitative imaging: Zhang et al.
- Bioinformatics for more effective healthcare: Kapelner et al.; Namdar et al.
- Innovative AI applications for patient safety: Chan et al.

## Papers Included in This Research Topic

In their work, Hart et al. developed seven machine learning algorithms based solely on personal health data from the Prostate, Lung, Colorectal, and Ovarian Cancer Screening Trial (PLCO), and compared them with 15 practicing physicians in stratifying endometrial cancer risk for 100 women. The results indicate that their random forest model achieves a testing AUC of 0.96, 2.5 times better at identifying above-average risk women with a 2-fold reduction in the false-positive rate. A novel concept named “Statistical Biopsy” was proposed for the first time.

Chen et al. reported their development of a deep-learning convolutional neural network (DCNN) for enhanced organ-at-risk (OAR) segmentation on cone beam computed tomography (CBCT), trained with forty post-operative head and neck cancer patients. The developed DCNN improved CBCT in terms of Hounsfield unit (HU) accuracy, image contrast, and OAR delineation accuracy.

Using a cohort of 100 prostate cancer patients, Jensen et al. demonstrated that their novel machine learning model can be used to quickly estimate the Pareto set of feasible dose objectives in cancer radiotherapy, which may directly accelerate the treatment planning process and indirectly improve final plan quality by allowing more time for plan refinement. Their model outperforms the existing machine learning techniques by utilizing optimization priorities and output initialization.

As a first attempt, Mistro et al. have demonstrated that knowledge models can be effectively used as teaching aid to bring inexperienced planners to a level close to experienced planners in fewer than 2 days. The proposed tutoring system can serve as an essential component in an AI ecosystem that will enable clinical practitioners to use knowledge-based planning effectively and confidently for personalized radiation treatment.

Based on 85 training cases and 15 test cases, Wang et al. have demonstrated a novel deep learning framework for pancreas stereotactic body radiation therapy (SBRT) planning, which can predict a fluence map for each beam, hence bypassing the lengthy inverse optimization process.

In their work, Barua et al. demonstrated that a Multivariate Functional Principal Component Analysis (MFPCA) approach can be used to characterize the temporal trajectories of mandibular subvolumes receiving radiation. Their work suggests that temporal trajectories of radiomics features derived from sequential pre- and post-RT CT scans correlate with radiotherapy-induced mandibular injury, which may be used to aid in earlier management of osteoradionecrosis, a major side-effect in radiation therapy of oropharyngeal cancer patients.

In a mini-review, Luo summarized three major approaches currently employed in predicting cervical cancer outcomes: statistical models, medical images, and machine learning, and discussed some of the challenges in making clinical outcome prediction more accurate, reliable, and practical.

Zhang et al. proposed a transfer learning-based prognostication model for overall survival in pancreatic ductal adenocarcinoma patients. The model achieved the area under the receiver operating characteristic curve (AUC) of 0.81, significantly higher than that of the traditional radiomics model of 0.54. Their result suggests that transfer learning-based models may significantly improve prognostic performance in typical small sample size medical imaging studies.

To evaluate the overall effectiveness of personalized medicine, Kapelner et al. introduced and discussed a novel R package called “Personalized Treatment Evaluator (PTE)” developed by them. They combined randomized comparative/controlled trial (RCT) data with a statistical model of the response to estimate outcomes under different treatment allocation protocols. Their PTE package can be used to evaluate personalization models in medicine as well as fields outside of medicine.

In their paper, Namdar et al. presented first a comprehensive review of AUC metric, and then proposed a modified version of AUC that takes confidence of the model into account and incorporated AUC into Binary Cross Entropy (BCE) loss function. They demonstrated the validity of the new concept on MNIST, prostate MRI, and brain MRI datasets.

In a review paper, Chan et al. discussed and summarized the various applications of machine learning approaches in machine-specific and patient-specific quality assurance (QA), a key component in safeguarding patient safety during the radiation treatment of cancer patients.

## Conclusions

Precision medicine is an evolving healthcare approach focused on tailoring medical decisions, treatments, practices, and products to individual patients based on their genetic, environmental, lifestyle, and other factors. In this Research Topic, eleven teams reported promising results from their experience in applying AI for precision medicine. Moving forward, we anticipate that more work needs to be done to eliminate biases in the AI models and make these models interpretable, therefore ultimately achieving the promise of precision medicine, i.e., delivering the right treatment to the right patient at the right time.

## Author Contributions

JD drafted the editorial. JD, TH, EC, JC, and FE-S revised and approved the final version. All authors contributed to the article and approved the submitted version.

## Funding

Research reported in this publication was supported by the National Institute of Biomedical Imaging and Bioengineering of the National Institutes of Health under Award Number R01EB022589, by the National Science Foundation under Award Number DMS 1918925, by the National Cancer Institute under Award Number 21X130F, and by the Department of Energy under Award Number DE-SC0021655 to JD. EC was supported by the grant NSF 19-500 under Award Number DMS 1918925 and 1922843 in years 2019-2022.

## Author Disclaimer

The content is solely the responsibility of the authors and does not necessarily represent the official views of those institutions.

## Conflict of Interest

The authors declare that the research was conducted in the absence of any commercial or financial relationships that could be construed as a potential conflict of interest.

## Publisher's Note

All claims expressed in this article are solely those of the authors and do not necessarily represent those of their affiliated organizations, or those of the publisher, the editors and the reviewers. Any product that may be evaluated in this article, or claim that may be made by its manufacturer, is not guaranteed or endorsed by the publisher.

